# Adrenal limb thickness is associated with metabolism profiles in patients with diabetes: A cross‐sectional study

**DOI:** 10.1111/1753-0407.13479

**Published:** 2023-09-26

**Authors:** Yingning Liu, Xiantong Zou, Wei Zhao, Xun Yao, Lexuan Wang, LingLi Zhou, Rui Zhang, Yingying Luo, Meng Li, Xiuying Zhang, Yu Zhu, Xiaoling Cai, Xianghai Zhou, Xueyao Han, Linong Ji

**Affiliations:** ^1^ Department of Endocrinology and Metabolism Peking University People's Hospital Beijing China; ^2^ Department of Radiology Peking University People's Hospital Beijing China; ^3^ School of Basic Medical Sciences Peking University Beijing China

**Keywords:** adrenal glands, diabetes mellitus, hypertension, metabolism, multidetector computed tomography

## Abstract

**Background:**

The association between adrenal size and metabolic profiles in patients with diabetes mellitus (DM) is unclear. This study was conducted to determine whether the adrenal thickness measured by computed tomography (CT) is correlated with the metabolic profiles of patients with DM.

**Methods:**

This was a cross‐sectional study including 588 Chinese hospitalized patients with DM without comorbidities or medications known to affect adrenal morphology or hormone secretion. Adrenal limb thickness was measured on unenhanced chest CT. Participants were stratified into tertiles according to their total adrenal limb thickness. Linear and logistic regression models were used to estimate the correlations.

**Results:**

After adjustment for sex and age, the adrenal thickness was positively associated with body mass index (BMI), waist circumference (WC), urinary albumin/creatinine ratio, and 24‐h urinary free cortisol (UFC) and negatively correlated with high‐density lipoprotein cholesterol. The sequential equation model (SEM) suggested UFC partially mediated the effect of adrenal limb thickness on WC by 12%. Adrenal thickness, but not UFC, was associated with a higher risk of existing hypertension (odds ratio [OR] = 3.78, 95% confidence interval [CI] 1.58, 9.02) and hyperlipidemia (OR = 2.76, 95% CI 1.03, 7.38), independent of age, gender, BMI, and WC.

**Conclusions:**

The adrenal thickness is independently associated with BMI, WC, cortisol levels, urinary albumin/creatinine ratio, hypertension, and dyslipidemia but not glycemic parameters in patients with diabetes. Our study encourages further studies to investigate the role of adrenal physiology in patients with diabetes.

## Graphical abstract

**Highlights**

- Adrenal thickness is independently associated with abdominal obesity, proteinuria, existing hypertension, and dyslipidemia.
- Cortisol may mediate the association between adrenal thickness and abdominal obesity.
- Adrenal physiology may have an essential role in the metabolism of patients with diabetes.

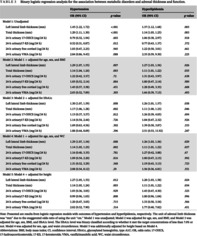

## 1 INTRODUCTION

Diabetes mellitus (DM) is a public health issue worldwide. Its prevalence has climbed to 10.9% in China.^1^ Multiple metabolic comorbidities, including hypertension, obesity, hyperlipidemia, fatty liver disease, and adrenal disorder, may develop concurrently or sequentially with DM. The connection between obesity and diabetes often involves mechanisms related to ectopic and visceral adiposity, both of which are closely associated with insulin resistance.^2^ Furthermore, the intricate interplay between diabetes and hypertension is notable. The dynamic interaction of elevated glucose and insulin levels is believed to have a synergistic effect on arterial stiffness and involved in the initial stages of hypertension's pathophysiology.^3^ Additionally, obesity contributes to insulin resistance, which in turn can lead to diabetes‐related hypertension. These interrelated factors highlight the multifaceted nature of obesity, diabetes, and hypertension and emphasize the need for a comprehensive understanding of their interactions.

The adrenal glands control a variety of vital physiological processes with endocrine secretory function.^4^ Adrenal adenomas with abnormal adrenal hormone production, such as primary aldosteronism and hypercortisolism, are closely relevant to dysregulated blood pressure, blood lipids, and blood glucose.^5^, ^6^, ^7^, ^8^ Adrenal morphological abnormity without aberrant adrenal functions, for example adrenal incidentaloma or adrenal hypertrophy, was regarded as lacking clinical significance for a long time. The significance of adrenal morphology to metabolic profiles has been unrecognized to some extent, until the last decades, evidence is accumulating on the association between adrenal size and metabolic disorders. The total adrenal volume was significantly higher in patients with type 2 diabetes when compared with patients without diabetes.^9^, ^10^ Askani et al also suggested the adrenal volume assessed by magnetic resonance imaging was an indirect marker of impaired glucose metabolism and hypothalamic–pituitary–adrenal (HPA) axis dysfunction.^11^ Given that the adrenal thickness has been hypothesized as the representation of HPA axis, the dysregulation of HPA axis may occur in patients with diabetes.^12^ Additionally, the reciprocal relationship between corticosteroids and diabetes was noted.^12^, ^13^, ^14^, ^15^ Further studies are required to elaborate the relationship between the metabolic profile and adrenal size, especially in participants with diabetes.

Computed tomography (CT) is a widely utilized imaging method to assess adrenal morphology in clinical practice^16^ due to its quick examination and highly reproducible results.^17^, ^18^ However, the current assessment of adrenal volume required sophisticated radiologists and advanced imaging technologies, for example 3D construction of CT images, which may limit the clinical utility. There is a clinical demand for simple parameters as a substitution of adrenal volume to evaluate the adrenal size. The concordance between volumetric and linear assessment was proved^17^ and a feasible method of measuring adrenal thickness had already been established.^19^ So far, two previous studies that adopted the adrenal limb thickness measurements to evaluate the gland dimensions have been conducted.^20^, ^21^ In the present study, we aimed to explore the relevance between adrenal linear measurement obtained on CT and the metabolic profile of individuals with diabetes using a hospital‐based cross‐sectional cohort in China.

## 2 MATERIALS AND METHODS

### 2.1 Study population

**FIGURE 1:**
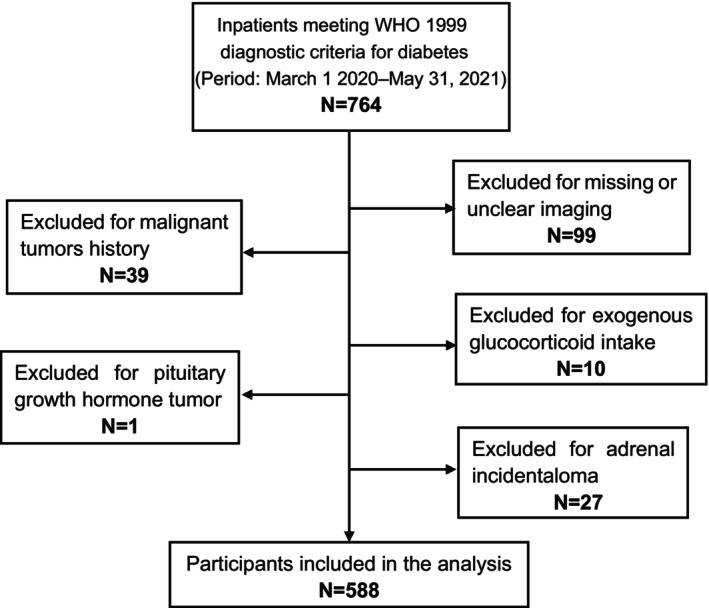
Study flow chart of participant inclusion and exclusion. WHO, World Health Organization.

This cross‐sectional, retrospective, single‐center study was conducted at the Department of Endocrinology and Metabolism, Peking University People's Hospital. Individuals with diabetes presenting to the inpatient ward of the Department of Endocrinology and Metabolism between March 2020 and May 2021 were recruited in our study initially. All the patients were hospitalized to manage their diabetes and improve their glycemic control without any requirement for other diseases. Diabetes was defined as the previous diagnosis with DM or newly diagnosed according to the Chinese Diabetes Society guidelines.^22^ Each hospitalized patient was required to receive an unenhanced chest CT scan before being admitted to the ward. The chest CT, in addition to the COVID‐19 nucleic acid test, was originally aimed to exclude any potential COVID‐19 infection, according to the hospital administration requirements. All participants admitted were confirmed absent from COVID‐19 infection before administration. Participants with unclear CT imaging or without both adrenal glands contained in the CT images were excluded. Further exclusion criteria were glucocorticoid treatments and comorbidities that may affect adrenal morphology and function including malignant disorders, primary aldosteronism, Cushing's syndrome, pituitary growth hormone tumors, and adrenal incidentaloma. A total of 764 inpatients initially met the enrolled criteria during the study period, as indicated in the patient flow chart shown in Figure 1. Ninety‐nine were excluded for missing or unclear CT imaging, 66 were excluded for malignant/adrenal tumors, and 11 were excluded for exogenous glucocorticoid intake or pituitary growth hormone tumor. Ultimately, 588 participants were included in our analysis. We conducted the 1 mg overnight dexamethasone suppression test on 25 out of the 588 participants who displayed clinical signs related to Cushing's syndrome. The results of this test effectively ruled out the presence of both Cushing's syndrome and subclinical Cushing's syndrome in all 25 of these participants. Fourteen of the 588 participants were found to have urinary free cortisol (UFC) higher than the normal range. Nine out of the 14 participants were confirmed without overt adrenal hyperfunction with negative 1 mg overnight dexamethasone suppression test. The other five participants were not tested for 1 mg overnight dexamethasone suppression test due to the possibility of developing diabetic ketoacidosis.

This study was conducted in compliance with the Declaration of Helsinki and was approved by the Ethics Committee of Peking University People's Hospital. The need for signed informed consent was waived.

### 2.2 Data collection

The Peking University People's Hospital Clinical Data Application Platform is a recently established database that integrates 18 hospital‐based data systems and contains medical information from more than 13 million individuals who had attended our hospital. With permission from the medical information center of Peking University People's Hospital, the data of the eligible participants were extracted from this database. Then we systematized the data using Python 3.9.12 followed by manual checking.

Medical history questionnaires and physical examinations were conducted and recorded by skilled medical professionals upon the first day of hospitalization. We also tracked whether hypoglycemia occurred during the whole course of diabetes from the medical records because long‐term hypoglycemia may trigger sympathetic nerve excitation and raise glucocorticoid levels. Height, weight, neck circumference, waist circumference, hip circumference, and blood pressure, sociodemographic characteristics, disease history, and medication history were obtained from the original medical records. Body mass index (BMI) was computed as weight in kilograms divided by height in meters squared.

All the laboratory assays were conducted in the clinical laboratory of Peking University People's Hospital. Fasting venous blood samples were collected in the morning. Fasting plasma glucose (FPG), lipid profiles, renal function, serum sodium, serum potassium, and serum albumin were determined utilizing an automatic biochemical analyzer (Hitachi LST008, Japan). Serum fasting C‐peptide was measured by the chemiluminescence method (Roche E411; Roche Diagnostics; Switzerland). Urinary albumin/creatinine ratio (ACR) was assessed using Roche C311 (Roche Diagnostics; Switzerland). The level of hemoglobin A1c (HbA1c) was quantified by automated high‐performance liquid chromatography according to a standard procedure (Premier Hb9210, USA).

Additionally, all participants were instructed to collect the 24‐h urine specimen during the hospitalization as a routine procedure of our inpatient Department of Endocrinology and Metabolism. The 24‐h collecting period started once the first void of urine was discarded on the first morning. All consecutive voids of urine were saved in a supplied container to examine the concentration of 24‐h urinary sodium, calcium, 17‐hydroxycorticosteroids (17‐OHCS), vanillylmandelic acid (VMA), 17‐ketosteroids (17‐KS), and UFC. Urine steroids were measured by homogeneous enzyme immunoassay. The calibrators of 17‐KS, 17‐OHCS, UFC, and VMA measurements can be traced back to the Cerilliant D‐063, Cerilliant C‐130, standard reference materials 921a of National Institute of Standards and Technology (USA), and national standard reference materials 09226 of China, respectively. The interassay and intraassay coefficient of variation of the cortisol metabolites measurements is <10.0%.

### 2.3 Definitions

Dyslipidemia was defined as total cholesterol ≥4.50 mmol/L, triglycerides ≥1.70 mmol/L, low‐density lipoprotein cholesterol ≥2.60 mmol/L, high‐density lipoprotein cholesterol (HDL‐C) <1.30 mmol/L for women or HDL‐C <1.00 mmol/L for men, or current antihyperlipidemic medication therapy, according to Chinese Diabetes Society guidelines.^22^ Hypertension was defined as a systolic blood pressure ≥140 mm Hg and/or a diastolic blood pressure ≥90 mm Hg, in addition to existing hypertension treatment. The homeostatic model assessment of insulin resistance 2 (HOMA2IR) and HOMA%2B were calculated using homeostasis model assessment based on paired fasting glucose and C‐peptide measurements^23^ (https://www.dtu.ox.ac.uk/homacalculator/).

### 2.4 CT examinations

The unenhanced chest CT scans were obtained in the supine position, using a 64‐slice multidetector scanner (Light Speed Volume CT; GE Healthcare, USA). The scanning started from the thoracic inlet to the upper abdomen within a breath‐hold following a deep inspiration. The acquisition parameters were as follows: 120 kV, 200 mAs, pitch 0.98:1, rotation time:0.6 s. A standard reconstruction algorithm was used and the reconstructed slice thickness was 1.25 mm. All the CT images were reserved in the Picture Archiving and Communications Systems (PACS) of Peking University People's Hospital.

### 2.5 CT measurement

**FIGURE 2:**
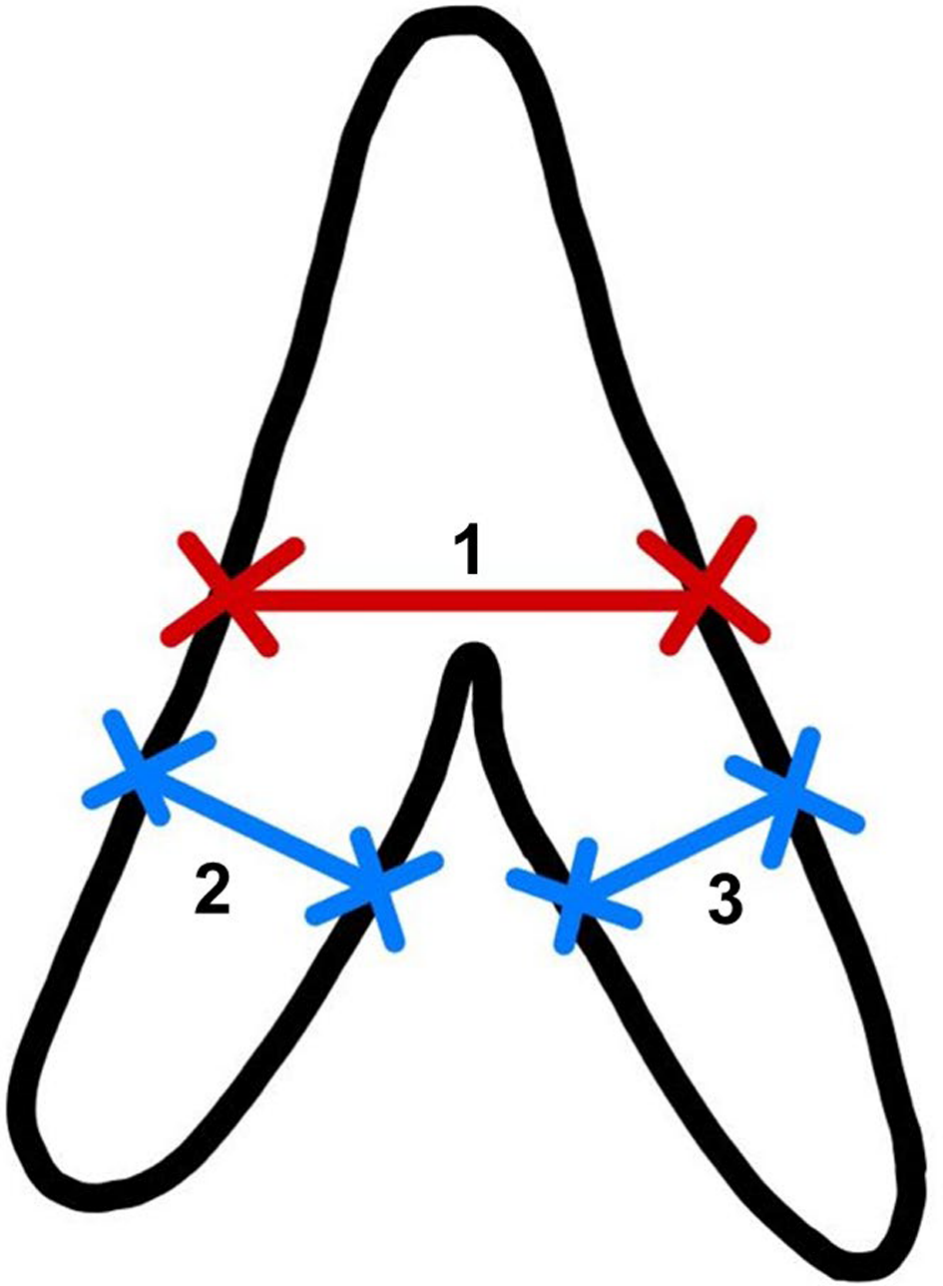
Schematic diagram of the adrenal thickness measurement of the body (1), the lateral limb (2), and the medial limb (3).

We retrospectively reviewed the CT scans on the PACS and the adrenal gland thickness assessment was performed by two observers: XY and YNL (a senior radiologist with more than 5 years of experience and a junior reader trained in abdominal imaging). The mediastinal window setting with 30 Hounsfield units (HU) level and 300 HU width was adopted for the observation. The measurement was made on the level that best shows each part and avoids any visible nodule. We followed the methodology outlined by Vincent et al for adrenal thickness measurement^19^: (a) the width of the adrenal gland was measured at its maximum width at the junction of the adrenal limbs and the body, perpendicular to the long axis of the adrenal body; and (b) the width of the adrenal limbs was measured at the maximum thickness of both the medial and the lateral limbs of the adrenal gland, perpendicular to their long axis (Figure 2). The measurements were obtained using the standard measurement function of the PACS by placing the cursor on the margins of the adrenal gland limbs. The average of two measurements was taken for each adrenal limb. The measurements were recorded in centimeters (cm). We found that about one fifth of the right lateral adrenal limbs were unable to be measured due to the interference of the liver. Therefore, we defined the “adrenal total thickness” as the sum of the other three limbs in our study, for it is more accessible in most clinical settings and thus a more meaningful marker.

### 2.6 Statistical analysis

Demographics and clinical characteristics according to adrenal total thickness categories were expressed as means ± standard deviation for continuous data with normal distribution, median (interquartile range) for continuous data with skewed distribution, and number (percentage) for categorical data. One‐way analysis of variance test was conducted to compare normally distributed continuous variables, and the Kruskal.–‐Wallis test was conducted to compare skewed distributed continuous variables between different groups. The chi‐square test was used to determine differences in categorical variables between groups.

Linear and logistic regression models were used to assess the relationships between metabolic profiles and adrenal total thickness. The adrenal total thickness served as the independent variable, and the metabolic profiles served as the dependent variable in the regression models after adjusting for gender and age. The systolic and diastolic blood pressure was analyzed with additional adjustment for the current treatment of hypertension, and the lipid profiles were analyzed with additional adjustment for the current use of statins. The false discovery rate correction for multiple comparisons, as described by Benjamini‐Hochberg,^24^ was applied to calculate the adjusted *p*‐value. An adjusted *p*‐value <0.05 was considered statistically significant.

SEM was employed to evaluate the mediating effect. The 24‐h UFC was the mediating variable, the adrenal total thickness was the independent variable, and the waist circumference was the dependent variable. The mediation analysis was conducted by STATA 16.0.

Logistic regression models were carried out to analyze the effects of adrenal profiles on the presence of hypertension and hyperlipidemia, respectively. Gender, age, and obesity might have potential effects on hypertension and hyperlipidemia, so we adjusted these confounders in different models. Model 1 was unadjusted, Model 2 was adjusted for age, sex, and BMI. In addition, because the disease control of DM could potentially influence the association between metabolic disorders and adrenal thickness or function, Model 3 was additionally adjusted for the HbA1c level. The HbA1c level was binary classified according to whether it met the target concentrations of <7.0% or not. Model 4 was adjusted for sex, age, and waist circumference. As the adrenal gland is one of the human tissue organs and has physiological variability, Model 5 was additionally adjusted for height based on Model 4.

All statistical analyses were performed with SPSS for Windows, vs 25.0 (SPSS, Inc., Chicago, IL, USA) unless specified. Significance was defined as a two‐sided *p* < .05.

## 3 RESULTS

### 3.1 The adrenal thickness of patients with diabetes

The histograms of adrenal gland limb thickness are presented in Figure S1. Each limb was on average wider on the left side compared to the opposite. The mean thickness of the medial limb was 0.38 (±0.10) cm on the right side and 0.49 (±0.15) cm on the left side. The lateral limbs had less difference between the two sides with a thickness of 0.33 (±0.10) cm and 0.36 (±0.12) cm, respectively. The mean value of “adrenal total thickness” defined by us was 1.23 (±0.28) cm.

### 3.2 Association between adrenal limb thickness and metabolic profiles

Patients were stratified into three subgroups based on tertiles of the adrenal total limb thickness: the adrenal total thickness of the three consecutive tertile groups was 0.73–1.08 cm, 1.09–1.29 cm, and 1.30–2.77 cm, respectively. The general features of the study participants in the three subgroups are summarized in Table 1. Participants with larger adrenal total thickness had a significantly higher BMI; greater neck circumference, waist circumference, and hip circumference; and a higher proportion of individuals with hypertension. The subgroups also differed significantly in terms of whether hypoglycemia occurred throughout the entire course of diabetes. There were also significant differences in gender, age, height, weight, and HDL‐C level among categories of the adrenal total thickness. Neither other plasma lipids nor glucose levels differed between these groups. However, serum creatinine, urinary ACR, 24‐h urinary 17‐KS, and 24‐h UFC elevated with the increase of adrenal total thickness.

**TABLE 1:** Clinical characteristics according to adrenal total thickness categories.

Characteristics	First tertile (0.73–1.08 cm)	Second tertile (1.09–1.29 cm)	Third tertile (1.30–2.77 cm)	Adjusted *p* value
*n*	148	149	149	
Adrenal total thickness, cm	0.97 ± 0.08	1.18 ± 0.06	1.55 ± 0.24	<.001
Men, *n* (%)	73 (49.3)	93 (62.4)	115 (77.2)	<.001
Age, years	54.3 ± 12.9	54.3 ± 13.2	57.2 ± 12.9	.160
Course of diabetes, years	10.7 ± 8.6	10.9 ± 8.7	11.4 ± 8.4	.830
Height, cm	163.44 ± 9.46	166.43 ± 8.92	166.98 ± 8.24	.005
Weight, kg	70.8 ± 12.9	74.8 ± 13.1	78.1 ± 14.3	<.001
BMI, kg/m^2^	26.44 ± 3.69	27 ± 3.5	27.98 ± 4.08	.009
Neck circumference, cm	37.8 ± 3.6	39.3 ± 6.8	39.8 ± 4.0	.010
Waist circumference, cm	93.9 ± 9.6	96.3 ± 9.7	99.7 ± 9.9	<.001
Hip circumference, cm	100.7 ± 6.9	101.5 ± 7.2	103.6 ± 8.3	.012
Hypertension, *n* (%)	62 (41.9)	81 (54.4)	103 (69.1)	<.001
Use of antihypertension drug, *n*	45	49	55	
Systolic blood pressure, mm Hg	135 ± 16	138 ± 19	137 ± 18	.489
Diastolic blood pressure, mm Hg	79 ± 14	79 ± 13	79 ± 12	.951
Hyperlipidemia, *n* (%)	106 (71.6)	111 (74.5)	122 (81.8)	.193
Use of statins, *n*	51	41	39	
Total cholesterol, mmol/L	4.62 ± 1.19	4.64 ± 1.57	4.44 ± 1.10	.522
Triglyceride, mmol/L	1.57 (1.02)	1.70 (1.40)	1.59 (1.39)	.689
HDL‐C, mmol/L	1.13 ± 0.29	1.11 ± 0.37	1.02 ± 0.20	.011
LDL‐C, mmol/L	2.83 ± 0.87	2.82 ± 1.02	2.74 ± 0.89	.734
Fasting C‐peptide, ng/mL	1.83 (1.23)	1.86 (1.37)	1.87 (1.14)	.658
FPG, mmol/L	8.5 ± 3.1	8.1 ± 2.8	8.0 ± 2.6	.602
HbA1c, %	9.1 ± 2.0	9.0 ± 2.0	9.3 ± 2.1	.691
HOMA2IR	4.55 (3.5)	4.55 (3.11)	4.51 (2.91)	.739
HOMA2%B, medi	115.7 (110.8)	129.1 (129.1)	117.3 (106.7)	. 677
Hypoglycemia, *n* (%)	26 (17.5)	36 (24.1)	16 (10.7)	.010
Serum sodium, mmol/L	140.8 ± 2.6	140.5 ± 2.5	140.4 ± 2.1	.527
Serum potassium, mmol/L	3.9 ± 0.3	4.0 ± 0.4	3.9 ± 0.4	.652
eGFR, ml/min	98.7 ± 18.8	96.4 ± 19.6	92.8 ± 21.8	.092
Albumin, g/L	41.2 ± 3.6	40.5 ± 4.1	40.0 ± 3.4	.031
AGR	1.62 ± 0.27	1.60 ± 0.27	1.55 ± 0.26	.155
Serum creatinine, umol/L	66.6 ± 18.1	72.0 ± 24.1	77.0 ± 32.4	.009
ACR, mg/g	7.1 (10.8)	9.6 (34.2)	9.6 (57.6)	.031
24 h urinary sodium, mmol/day	165 ± 64	179 ± 83	182 ± 88	.263
24 h urinary calcium, mmol/day	5.3 ± 2.7	5.5 ± 3.3	5.2 ± 2.9	.777
24 h urinary 17‐OHCS, mg/24 h	5.75 (2.73)	5.40 (2.80)	5.50 (2.90)	.712
24 h urinary17‐KS, mg/24 h	7.3 ± 5.2	8.7 ± 5.8	10.0 ± 7.0	.009
24 h urinary free cortisol, ug/24 h	160.4 (79.9)	169.0 (84.0)	204.3 (107.1)	<.001
24 h urinary VMA, mg/24 h	5.75 (2.6)	5.60 (2.10)	5.50 (2.20)	.940

*Note*: Data were expressed as means ± SD for continuous variables with normal distribution, median (interquartile range) for continuous variables with skewed distribution, and number (percentage) for categorical variables. One‐way analysis of variance test for normally distributed continuous variables, Kruskal–Wallis test for skewed distributed continuous variables, and chi‐square test for categorical variables were conducted to determine the differences between groups according to the adrenal total thickness. Hypoglycemia was defined as whether hypoglycemia occurs during the whole course of diabetes.

Abbreviations: ACR, albumin‐to‐creatinine ratio; AGR, albumin to globulin ratio; BMI, body mass index; eGFR, estimated glomerular filtration rate; FPG, fasting plasma glucose; HbA1c, glycosylated hemoglobin, type A1C; HDL‐C, high‐density lipoprotein cholesterol; LDL‐C, low‐density lipoprotein cholesterol; VMA, vanillylmandelic acid; 17‐OHCS, 17‐hydroxycorticosteroids; 17‐KS, 17‐ketosteroids.

Table 2 shows the linear and logistic regression analysis results between adrenal total thickness and metabolic profiles. Significant associations between adrenal total thickness and anthropological measurements, including BMI, waist circumference, and hip circumference, were observed after adjustment for age and sex. The fasting C‐peptide was also positively correlated with adrenal total thickness, although no significant correlations were found between adrenal total thickness and HbA1c and FPG. The concentration of HDL‐C was significantly and negatively associated with the adrenal total thickness. For parameters determined in 24‐h urine collections, urinary sodium, 17‐KS, and UFC were positively associated with adrenal total thickness. Other parameters significantly associated with the adrenal total thickness after adjustment were serum sodium, serum album, serum albumin/globulin, and urinary ACR. No discernible correlation between adrenal total thickness and the incidence of hypoglycemia is evident (*p* = .085).

**TABLE 2:** Associations between demographics, metabolic profiles, and adrenal total thickness.

Variables	Estimate (β)	Adjusted *p* value
Height (continuous, kg/m^2^)	1.404	.273
Weight (continuous, kg/m^2^)	9.844	<.001
BMI (continuous, kg/m^2^)	3.08	<.001
Waist circumference (continuous, cm)	8.18	<.001
Hip circumference (continuous, cm)	4.79	<.001
Systolic blood pressure (continuous, mm Hg)	4.78	.223^†^
Diastolic blood pressure (continuous, mm Hg)	−1.17	.782^†^
Total cholesterol (continuous, mmol/L)	−0.10	.871^‡^
Triglyceride (continuous, mmol/L)	0.06	.273^‡^
HDL‐C (continuous, mmol/L)	−0.06	.004^‡^
LDL‐C (continuous, mmol/L)	0.002	.974^‡^
Fasting C‐peptide (continuous, ng/mL)	0.15	.041
FPG (continuous, mmol/L)	−0.07	.958
HbA1c (continuous, %)	0.23	.772
HOMA2IR (continuous)	0.04	.740
HOMA2%B (continuous)	0.01	.953
Serum sodium (continuous, mmol/L)	−1.20	.01
Serum potassium (continuous, mmol/L)	−0.002	.978
eGFR (continuous, ml/min)	−4.39	.228
Albumin (continuous, g/L)	−2.05	.004
AGR (continuous)	−0.19	<.001
Serum creatinine (continuous, umol/L)	9.22	.051
ACR (continuous, mg/g)	0.44	.004
24 h urinary calcium (continuous, mmol/day)	0.19	.842
24 h urinary sodium (continuous, mmol/day)	33.75	.04
24‐h urinary 17‐OHCS (continuous, mg/24 h)	−0.02	.757
24‐h urinary17‐KS (continuous, mg/24 h)	3.57	<.001
24‐h urinary free cortisol (continuous, ug/24 h)	0.14	.004
24‐h urinary VMA (continuous, mg/24 h)	−0.01	.771
Hypoglycemia (0/1)	0.348	.085

*Note*: Results from linear and logistic regression analysis using the adrenal total thickness as the independent variable and the metabolic profiles as the dependent variable. All models were adjusted for age and sex. The adjusted *p* value was calculated by false discovery rate correction. An adjusted *p* value <.05 was considered statistically significant.

Abbreviations: ACR, albumin‐to‐creatinine ratio; AGR, albumin to globulin ratio; BMI, body mass index; EGFR, estimated glomerular filtration rate; FPG, fasting plasma glucose; HbA1c, glycosylated hemoglobin, type A1C; HDL‐C, high‐density lipoprotein cholesterol; LDL‐C, low‐density lipoprotein cholesterol; 17‐OHCS, 17‐hydroxycorticosteroids; 17‐KS, 17‐ketosteroids; VMA, vanillylmandelic acid.

^†^ Additionally adjusted for the use of antihypertension drug or not.

^‡^ Additionally adjusted for use of statins or not.

We also performed SEM analysis to discover whether the cortisol level mediates the association between the adrenal total thickness and waist circumference. A diagrammatic representation of SEM is presented in Figure S2. The analysis demonstrated that the positive correlation between adrenal total thickness and waist circumference was partially mediated by the 24‐h UFC (mediation effect 12%).

### 3.3 Association between metabolic disorders and adrenal thickness and function

The results of the adrenal profiles in the prediction of hypertension and hyperlipidemia after logistic regression analysis were detailed in Table 3. The adrenal total thickness and the left lateral limb thickness, which we believed to have the least variation in morphology among the four limbs, were presented. The results of the other three adrenal limbs were shown in the Supplementary Table S1. Increased adrenal thickness, both the adrenal limb thickness and adrenal total thickness, was associated with increased risk of hypertension, even after adjustment for age, sex, BMI, and HbA1c level or adjustment for age, sex, waist circumference, and height. There was an association between urinary 17‐KS and hypertension in crude; however, this association was attenuated and became insignificant after being adjusted for the confounders.

**TABLE 3:** Binary logistic regression analysis for the association between metabolic disorders and adrenal thickness and function.

	Hypertension		Hyperlipidemia	
	OR (95% CI)	*p* value	OR (95% CI)	*p* value
Model 1: Unadjusted				
Left lateral limb thickness (mm)	1.45 (1.22, 1.72)	<.001	1.37 (1.12, 1.68)	.003
Total thickness (mm)	1.20 (1.11, 1.30)	<.001	1.14 (1.05, 1.25)	.003
24‐h urinary 17‐OHCS (mg/24 h)	0.79 (0.32, 1.94)	.603	1.06 (0.38, 2.97)	.918
24‐h urinary17‐KS (mg/24 h)	0.52 (0.31, 0.87)	.012	0.77 (0.43, 1.37)	.372
24‐h urinary free cortisol (ug/24 h)	1.03 (0.47, 2.25)	.945	1.22 (0.50, 3.01)	.661
24‐h urinary VMA (mg/24 h)	2.84 (0.86, 9.43)	.088	2.35 (0.59, 9.30)	.225
Model 2: Model 1 + adjusted for age, sex, and BMI				
Left lateral limb thickness (mm)	1.29 (1.07, 1.55)	.007	1.27 (1.03, 1.56)	.026
Total thickness (mm)	1.14 (1.04, 1.24)	.003	1.11 (1.01, 1.22)	.035
24‐h urinary 17‐OHCS (mg/24 h)	1.22 (0.42, 3.57)	.72	1.31 (0.43, 3.97)	.638
24‐h urinary17‐KS (mg/24 h)	1.05 (0.52, 2.14)	.884	1.00 (0.47, 2.14)	.989
24‐h urinary free cortisol (ug/24 h)	1.43 (0.57, 3.58)	.451	1.28 (0.49, 3.35)	.608
24‐h urinary VMA (mg/24 h)	2.03 (0.52, 7.89)	.305	1.66 (0.39, 7.15)	.493
Model 3: Model 2 + adjusted for HbA1c				
Left lateral limb thickness (mm)	1.30 (1.07, 1.59)	.008	1.26 (1.01, 1.57)	.038
Total thickness (mm)	1.17 (1.06, 1.28)	.002	1.11 (1.00, 1.23)	.046
24‐h urinary 17‐OHCS (mg/24 h)	1.15 (0.37, 3.57)	.812	1.26 (0.39, 4.05)	.694
24‐h urinary17‐KS (mg/24 h)	1.14 (0.54, 2.44)	.726	1.04 (0.47, 2.32)	.926
24‐h urinary free cortisol (ug/24 h)	1.69 (0.63, 4.56)	.302	1.39 (0.50, 3.87)	.533
24‐h urinary VMA (mg/24 h)	1.88 (0.44, 8.09)	.396	2.51 (0.53, 11.92)	.247
Model 4: Model 1 + adjusted for age, sex, and WC				
Left lateral limb thickness (mm)	1.29 (1.07, 1.54)	.008	1.26 (1.03, 1.56)	.029
Total thickness (mm)	1.14 (1.05, 1.25)	.002	1.11 (1.01, 1.22)	.037
24‐h urinary 17‐OHCS (mg/24 h)	1.16 (0.40, 3.35)	.781	1.27 (0.42, 3.88)	.67
24‐h urinary17‐KS (mg/24 h)	1.09 (0.54, 2.20)	.816	1.00 (0.47, 2.15)	.992
24‐h urinary free cortisol (ug/24 h)	1.31 (0.52, 3.29)	.568	1.19 (0.45, 3.13)	.723
24‐h urinary VMA (mg/24 h)	2.08 (0.54, 8.12)	.29	1.56 (0.36, 6.81)	.551
Model 5: Model 4 + adjusted for height				
Left lateral limb thickness (mm)	1.27 (1.05, 1.53)	.012	1.28 (1.03, 1.58)	.024
Total thickness (mm)	1.14 (1.05, 1.24)	.003	1.11 (1.01, 1.22)	.034
24‐h urinary 17‐OHCS (mg/24 h)	1.05 (0.36, 3.05)	.929	1.45 (0.47, 4.50)	.523
24‐h urinary17‐KS (mg/24 h)	0.99 (0.96, 1.03)	.808	1.00 (0.96, 1.04)	.996
24‐h urinary free cortisol (ug/24 h)	1.20 (0.47, 3.05)	.715	1.33 (0.50, 3.54)	.566
24‐h urinary VMA (mg/24 h)	2.02 (0.52, 7.90)	.314	1.77 (0.40, 7.82)	.451

*Note*: Presented are results from logistic regression models with outcomes of hypertension and hyperlipidemia, respectively. The unit of adrenal limb thickness was “mm” due to the exaggerated odds ratio of using the unit “cm.” Model 1 was unadjusted; Model 2 was adjusted for age, sex, and BMI; and Model 3 was adjusted for age, sex, BMI, and HbA1c level. The HbA1c level was binary classified according to whether it met the target concentrations of less than 7.0% or not. Model 4 was adjusted for sex, age, and waist circumference. Model 5 was additionally adjusted for height based on Model 4.

Abbreviations: BMI, body mass index; CI, confidence interval; HbA1c, glycosylated hemoglobin, type A1C; OR, odds ratio; 17‐OHCS, 17‐hydroxycorticosteroids; 17‐KS, 17‐ketosteroids; VMA, vanillylmandelic acid; WC, waist circumference.

A similar pattern could be observed in hyperlipidemia. The increased adrenal total thickness and left lateral limb thickness were also associated with higher risk of hyperlipidemia and the relationships persisted after all the adjustment. However, once adjusted for the confounders, the association between the other three adrenal limb thickness and hyperlipidemia became nonsignificant. Besides, the adrenal function parameters (urinary 17‐OHCS, 17‐KS, UFC, and VMA) were not associated with hyperlipidemia in both unadjusted and adjusted models.

## 4 DISCUSSION

This hospital‐based cross‐sectional study in China revealed that adrenal total thickness was significantly associated with multiple metabolic profiles and diseases in participants with diabetes. Patients with increased adrenal thickness were characterized by higher BMI, waist circumference, serum creatine, and urinary ACR levels and lower HDL‐C and serum album values and were more likely to have hypertension. Besides, metabolites of cortisol were positively associated with adrenal thickness and may act as the mediator in the interaction between adrenal limb thickness and abdominal obesity. In particular, the increased adrenal total thickness can perform as an independent predictor for the presence of hypertension and hyperlipidemia.

Our findings were consistent with the previous studies conducted in the general population that there was a positive correlation between obesity, particularly abdominal obesity, and adrenal volume. Askani et al also put forth the notion that adrenal volume exhibits an association with variables such as BMI, waist circumference, triglyceride levels, and hypertension even after making adjustments.^11^This observation was made within a population‐based cohort that included both individuals with and without diabetes. Another study carried out by Gurun et al^25^also demonstrated a positive correlation between adrenal gland volumes (AGVs) and waist circumference, as well as BMI, in patients devoid of endocrine disorders like diabetes. However, Godoy‐Matos et al^10^ indicated that although visceral fat correlated with adrenal volume among obese individuals, this correlation was not evident within the diabetic subgroup. According to a recent prospective study, obese individuals had considerably larger AGVs than overweight participants and comparably healthy volunteers.^26^ Our study added to evidence that the positive association between central obesity and adrenal thickness existed not only in the general population but also participants with diabetes, the research results in this study may not specific to diabetes population.

Our study also demonstrated a positive association between 24‐h UFC and adrenal thickness. The 24‐h UFC is a cumulative index of circulating free (biologically active) cortisol and has long been regarded as the benchmark of integrated cortisol production. In individuals with proven ACTH‐dependent Cushing's syndrome, adrenal limb thickness paralleled with 24‐h UFC.^27^ However, whether adrenal size within the normal range was still associated with corticosteroid function was unknown. In a small sampled cohort, Golden et al demonstrated that the adrenal gland volume strongly correlated with adrenal function assessed by total daily salivary cortisol and dexamethasone‐suppressed salivary cortisol.^28^ Our study extended the field by adding evidence that adrenal size was associated with excess cortisol secretion in patients with diabetes.

Our results indicate that the relationship between adrenal thickness and abdominal obesity is partially intermediated by cortisol levels. However, we are unable to discern a causative effect from the current data. Given that the hyperactivity of the HPA axis in obesity is well established,^29^ increased adrenal thickness and elevated cortisol levels have been hypothesized as the representation of HPA axis dysregulation. Physiologically, 11β‐hydroxysteroid‐dehydrogenase type 1 (11β‐HSD‐type 1) converts inactive cortisone into active cortisol. The 11beta‐HSD1‐mediated dysregulation of cortisol metabolism in obese individuals is tissue specific. Reactivation of cortisone to cortisol is inhibited in the liver but enhanced in adipose tissue.^30^, ^31^ The altered activity of 11β‐HSD type 1 as well as other cortisol metabolism‐related enzymes induces an increase in cortisol metabolic rate.^41^ Thus, obesity activates the metabolic clearance of cortisol and, subsequently activate the HPA axis.^32^ The stimulation of the HPA axis encourages adrenal enlargement via the nutritive effect of ACTH. On the other hand, the dysregulation of HPA axis may cause glucocorticoid secretion as well as adrenal hyperplasia concurrently. Because glucocorticoids prompt the maturation of preadipocytes to adipocytes, elevated cortisol levels have been causally linked to fat accumulation and weight gain, leading to obesity ultimately.^33^

Data on glucose metabolism and adrenal morphology in individuals with diabetes are scarce and discordant: Askani et al identified the increased AGV as an indirect marker of impaired glucose metabolism.^11^ However, Serifoglu et al^9^ observed a negative association between glycemic status and AGV in type 2 diabetes patients. Our data suggested that glycemic parameters, except for the fasting C‐peptide, were not significantly correlated with adrenal thickness. Additional adjustments disclosed that BMI was the confounder in the relation between fasting C‐peptide and adrenal thickness. HOMA2IR or HOMA2%B can be more objective parameter to evaluate participant's glucose metabolism. Our results indicated that the adrenal limb thickness was neither associated with insulin resistance nor the beta cell function. In our study, nearly all patients were on pharmacological treatment of diabetes and the original beta cell function and insulin resistance may be altered. It would be worth to investigate the association between adrenal thickness and HOMA2IR and HOMA2%B in drug‐naive patients in further studies.

The present study also discovered an intriguing relationship between increasing adrenal thickness and existing hypertension, independent of BMI and waist circumference in patients with diabetes. Our finding further supported the previous data showing an association between increased AGVs and hypertension by Askani et al.^11^ It has been proved that adrenal hormones such as cortisol, catecholamine, and aldosterone can elevate blood pressure. Therefore, we investigated the concentration of cortisol and catecholamine metabolic products in urine. Neither the UFC nor urinary VMA was associated with hypertension in our study. In this light, we assumed that aldosterone level, which we were unable to quantify, is the intermediary between adrenal thickness and hypertension. In the current study, however, the increased adrenal thickness was found to be positively associated with 24‐h urinary sodium and inversely associated with serum sodium, which indicated an increase in sodium excretion. This is contrary to our hypothesis because aldosterone raises blood pressure primarily by prompting sodium reabsorption. Given that participants were not requested to discontinue the administration of antihypertensive drugs, these conflicting results might be explained at least partially by the application of diuretics. Thus, we highlighted a need for more studies to clarify the intermediate of increased adrenal thickness on hypertension and may further figure out the possible cause of resistant hypertension in diabetes.

Additionally, we observed that patients with wider adrenal thickness had elevated levels of urinary ACR and serum creatinine, both of which indicated poorer renal function. Probably, the reduced albumin and albumin‐to‐globulin ratio levels in individuals with increased adrenal thickness were also caused by the decline in renal function. In addition to the detrimental impact that concomitant hypertension exerts on the kidney, aldosterone may also impair glomerular filtration via other mechanisms. Zhang et al reported that even within the normal range, higher cortisol level was related to the development of microalbuminuria.^34^ Therefore, further research should be conducted to explore the potential mechanism underlying adrenal hypertrophy on renal dysfunction.

Adrenal limb thickness may play an important role in the adrenal function and other metabolic disorders in patients with diabetes. Also, for diabetes participants with uncontrolled obesity, hypertension, and hyperlipidemia, the adrenal limb thickness on CT scans could be examined to exclude the possible effect of adrenal function on metabolic profiles. Our study encourages further studies to investigate the impact of adrenal size and function on metabolic disorders.

Our study strengths include subject numbers and the initial evidence of the independent predictive value of adrenal morphology on the metabolism status in hospital‐based DM population. Besides strengths, our work presents certain limitations. First, the primary aldosteronism, which typically involves microadenomas and would maintain normal adrenal thickness, might have a high prevalence in this cohort. It is important to clarify that our study did not evaluate microaldosterone‐producing adenomas due to the absence of aldosterone level assessment and contrast‐enhanced scans. We admitted that the relationship between adrenal thickness and aldosterone levels represents a significant confounding factor and limitation of our study. In new‐onset type 2 diabetes patients with hypertension, primary aldosteronism (PA) was unexpectedly prevalent according to recent research.^35^ This finding suggests that autonomic hypersecretion of aldosterone in diabetes is frequent. Degenhart et al also found that PA patients had greater adrenal volumes than normal controls.^36^ According to a study adopting linear measurement identical to our approach, adrenal widths in PA patients with bilateral adrenal hyperplasia are substantially larger than in normal controls.^37^ In addition, a pilot study also indicated that adrenal volume was linked with plasma levels of glucocorticoids and aldosterone in healthy volunteers, although this conclusion applies to the most experienced observer only.^38^ Thus, it is quite likely that the aldosterone level plays a role in the relationship between adrenal thickness and hypertension. Second, because of the initial cross‐sectional design, we cannot infer the cause‐and‐effect between adrenal thickness and adrenal function and metabolic profiles. Additional longitudinal studies were necessary for the causality of adrenal morphology and metabolic disorders. Third, only a few patients in our cohort did the 1 mg overnight or 2 mg 48‐h dexamethasone suppression test, which was regarded as the most sensitive method to evaluate autonomous cortisol secretion in adrenal disease.^39^Nevertheless, the 24‐h UFC test we adopted also demonstrated high sensitivity and specificity for the diagnosis and evaluation, especially in patients with the typical features of hypercortisolism.^40^ Lastly, as compared to mass spectrometry, the homogeneous enzyme immunoassay for cortisol metabolites was not a state‐of‐the‐art measurement of cortisol, because urine may contain cross‐reacting substances that cause interference in immunoassays. However, studies have already proved that the immunoassay and mass spectrometry of UFC presented a similar diagnostic accuracy.^41^, ^42^

## 5 CONCLUSION

In conclusion, this hospital‐based study indicated that the adrenal total thickness measured by CT is independently associated with BMI, waist circumference, cortisol levels, hypertension, and dyslipidemia in patients with diabetes. Cortisol and its metabolite levels may mediate the associations between adrenal thickness and abdominal obesity. Adrenal physiology, particularly in connection with adrenal thickness, may have an essential role in the metabolism of patients with diabetes.

## AUTHOR CONTRIBUTIONS

Yingning Liu conducted the study, collected and analyzed the data, and drafted the manuscript. Xiantong Zou designed the study, analyzed the data, and edited the manuscript. Xiuying Zhang and Linong Ji designed the study and revised the manuscript. Wei Zhao contributed to the data collection and analysis. Xun Yao guided the methodology and collected data. Lexuan Wang, Yingying Luo, Meng Li, LingLi Zhou, Rui Zhang, Xiuying Zhang, Yu Zhu, Xiaoling Cai, and Xianghai Zhou contributed to data collection. All authors approved the final version of the manuscript.

## FUNDING INFORMATION

Beijing Nova Cross program (Z211100002121169 to Xiantong Zou) and Beijing Nova Program of Science and Technology (Z191100001119026 to Xiantong Zou), the National Natural Science Foundation of China (81970708 to Linong Ji), Beijing Science and Technology Commission (Z201100005520013 to Linong Ji), Clinical Medicine Plus X—Young Scholars Project, Peking University, the Fundamental Research Funds for the Central Universities (PKU2022LCXQ004 to Xiantong Zou) Peking University People's Hospital Scientific Research Development Funds (RDH2021‐10 to Xiantong Zou).

## CONFLICT OF INTEREST STATEMENT

The authors declare that they have no competing interests.

## Supporting information


**Data S1.** Supplementary Information.

## Data Availability

The data sets generated and analyzed during the current study are not publicly available due to human resource regulations of China, but are available from the corresponding author upon reasonable request. Linong Ji is the guarantor of this work and, as such, had full access to all the data in the study and takes responsibility for the integrity of the data and the accuracy of the data analysis.
