# Supplementary material for: Adrenal limb thickness is associated with metabolism profiles in patients with diabetes: A cross‐sectional study
Source: J Diabetes. 2023 Sep 26;16(2):e13479. doi: 10.1111/1753-0407.13479 (PMC10859314; doi:10.1111/1753-0407.13479)
Supplement: Supplementary file 1 — Data S1. Supplementary Information. [file JDB-16-e13479-s001.docx]

Supplementary material

Figure S1: Histogram derived of adrenal thickness measurement data from 588 patients with diabetes.
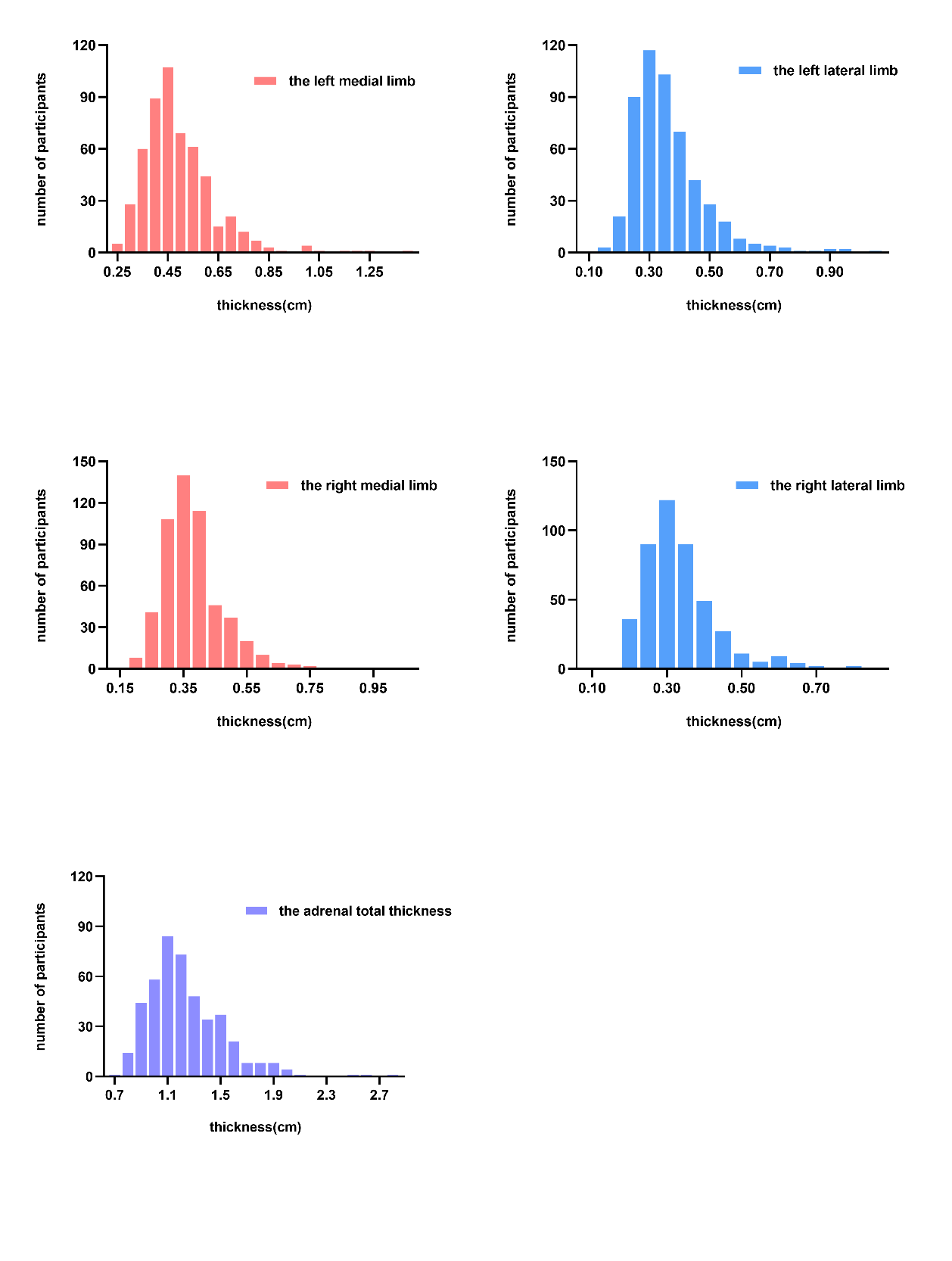


The adrenal total thickness was defined as the sum of the adrenal left medial limb, left lateral limb, and right medial left. Thickness was recorded in centimeters (cm).

Figure S2: Structural Model for the mediating effect of the cortisol level between adrenal thickness and waist circumference.


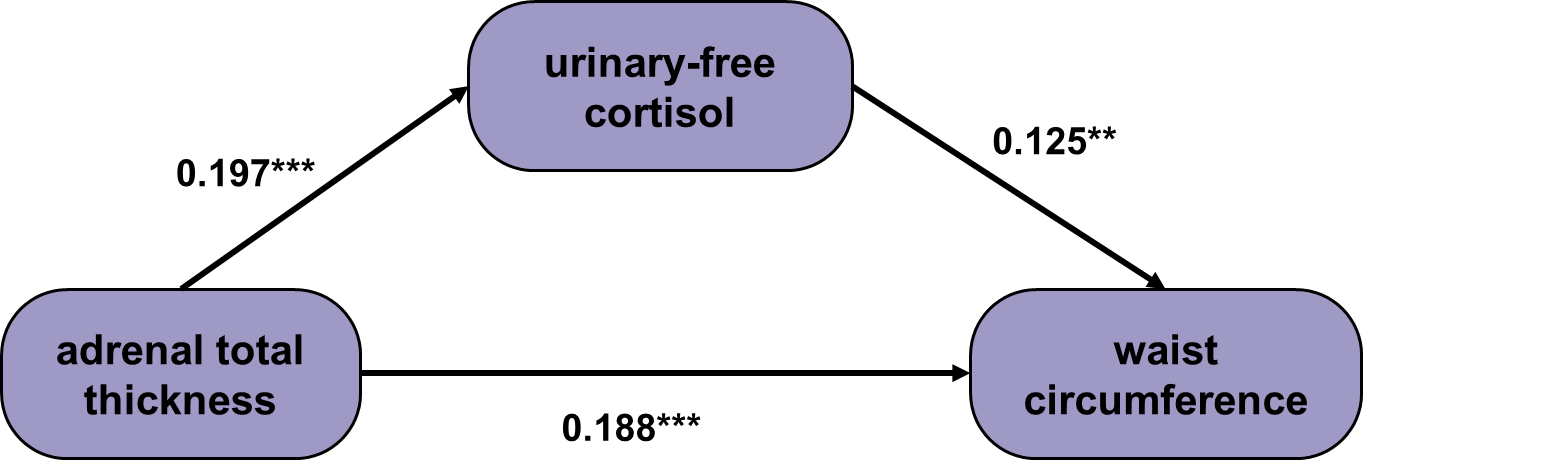


The structural model shows pathway coefficients between adrenal total thickness, urinary-free cortisol, and waist circumference. All indirect and direct pathways were significant. *** p= <0.001, ** p= <0.05

Table S1: Binary logistic regression analysis for the association between metabolic disorders and adrenal thickness.

| ***Model 1：unadjusted.*** | | | | |
| --- | --- | --- | --- | --- |
|  | ***hypertension*** | | ***hyperlipidemia*** | |
|  | **OR(95%CI)** | **p** | **OR(95%CI)** | **p** |
| **Left Medial limb thickness(cm)** | **6.47(1.80, 23.27)** | **0.004** | **3.25(0.76, 13.98)** | **0.113** |
| **Right Lateral limb thickness(cm)** | **179.80(17.76, 1819.72)** | **<0.001** | **32.41(2.46, 426.96)** | **0.008** |
| **Right Medial limb thickness(cm)** | **176.18(21.92, 1415.69)** | **<0.001** | **14.99(1.55, 145.16)** | **0.019** |
| ***Model 2： Model 1 + adjusted for age, sex, and BMI*** | | | | |
|  | ***hypertension*** | | ***hyperlipidemia*** | |
|  | **OR(95%CI)** | **p** | **OR(95%CI)** | **p** |
| **Left Medial limb thickness(cm)** | **4.09(0.99, 16.98)** | **0.052** | **2.08(0.46, 9.36)** | **0.342** |
| **Right Lateral limb thickness(cm)** | **29.22(2.53, 337.18)** | **0.007** | **12.08(0.84, 172.95)** | **0.067** |
| **Right Medial limb thickness(cm)** | **12.88(1.35, 122.70)** | **0.026** | **2.24(0.21, 23.86)** | **0.505** |
| ***Model 3： Model 2 + adjusted for HbA1c*** | | | | |
|  | ***hypertension*** | | ***hyperlipidemia*** | |
|  | **OR(95%CI)** | **p** | **OR(95%CI)** | **p** |
| **Left Medial limb thickness(cm)** | **5.15(1.10, 24.14)** | **0.038** | **2.32(0.46, 11.61)** | **0.306** |
| **Right Lateral limb thickness(cm)** | **31.80(2.06, 490.49)** | **0.013** | **10.81(0.60, 194.70)** | **0.106** |
| **Right Medial limb thickness(cm)** | **13.61(1.15, 160.99)** | **0.038** | **1.13(0.09, 13.73)** | **0.925** |
| ***Model 4： Model 1 + adjusted for age, sex, and WC*** | | | | |
|  | ***hypertension*** | | ***hyperlipidemia*** | |
|  | **OR(95%CI)** | **p** | **OR(95%CI)** | **p** |
| **Left Medial limb thickness(cm)** | **5.13(1.26, 20.91)** | **0.022** | **2.27(0.51, 10.10)** | **0.28** |
| **Right Lateral limb thickness(cm)** | **27.48(2.36,319.78)** | **0.008** | **12.43(0.85, 181.48)** | **0.065** |
| **Right Medial limb thickness(cm)** | **10.94(1.13,106.20)** | **0.039** | **2.02(0.18, 22.30)** | **0.565** |
| ***Model 5： Model4 + adjusted for height*** | | | | |
|  | ***hypertension*** | | ***hyperlipidemia*** | |
|  | **OR(95%CI)** | **p** | **OR(95%CI)** | **p** |
| **Left Medial limb thickness(cm)** | **4.98(1.22, 20.31)** | **0.025** | **2.38(0.53, 10.64)** | **0.255** |
| **Right Lateral limb thickness(cm)** | **24.43(2.04, 293.15)** | **0.012** | **12.61(0.88, 180.72)** | **0.062** |
| **Right Medial limb thickness(cm)** | **12.45(1.24, 124.56)** | **0.032** | **1.95(0.18, 21.36)** | **0.585** |
| **Note:** Presented are results from logistic regression models with outcomes of hypertension and hyperlipidemia, respectively. Model 1 was unadjusted, Model 2 was adjusted for age, sex, and BMI, and Model 3 was adjusted for age, sex, BMI and HbA1c level. The HbA1c level was binary-classified according to whether it met the target concentrations of less than 7.0% or not. Model 4 was adjusted for sex, age and waist circumference. Model 5 was additionally adjusted for height based on Model 4.  **Abbreviations：**17-OHCS, 17-hydroxycorticosteroids;17-KS, 17-ketosteroids; VMA, vanillylmandelic acid; HbA1c, glycosylated hemoglobin, type A1C**.** | | | | |
